# Intra-vaginal Respiration Recommended in Some Cases of Parturition Where the Child’s Life Is in Danger from Pressure on the Cord

**Published:** 1849-10

**Authors:** M. McCulloch


### OBSTETRICS.

*Intra-vaginal Respiration recommended in some cases of Parturition where the Child's Life is in Danger from Pressure on the Cord.* By M. McCulloch, M. D.—The writer desires to make known a new mode of practice, by which he thinks that the amount of infant mortality will be lessened in many cases in which death, from pressure on the cord, is otherwise inevitable. "If we take the average of French, German, and British practice, we find by the most authentic statistics that danger to the life of the child from this cause occurs in breech presentations once in 53 labors; in presentations of the superior extremities, after turning, once in 261; and where the cord presents, once in 245 cases. Thus we have in 649 labors 12 presentations of the breech, 7 of the inferior extremities, 2½ of the superior extremities, and 2½ of the cord, occasioning a loss of life at birth of about two per cent. of all children born. This great mortality I think I have succeeded in diminishing in my own practice in three footling cases within the last eight months, by enabling the child to breathe while the head was still within the cavity of the pelvis, after the pulsation in the cord had been several minutes extinct. And this novel state of existence was maintained by keeping the child's mouth open with my finger, and the perineum expanded to allow air to have free access into the vagina. In future, I shall endeavor to accomplish this object with greater certainty by introducing into the mouth the end of the largest-sized gum-elastic male catheter, with several perforations in it, and continue as before to allow as much air as possible to approach the face and nostrils. In all cases of malposition in which the practice I have suggested becomes desirable, the chance of respiration being established will be greater, if the means are used before the circulation becomes feeble in the umbilical vessels; and if the child is born alive in the absence of pulsation in them, it may fairly be conceded that we have been successful in our efforts to preserve life. Some cases may occur where a small-sized male catheter, with a suitable curve, might be used with advantage as a tracheal pipe. I need scarcely add that the infant's body should be kept warm, gentle traction employed, and all the other ordinary means of exciting contraction of the uterus used until the delivery is completed.

"If the practice I have here recommended is found to answer in other hands as it has in my own, something will have been achieved in obstetric science, in circumstances where the most skilful have hitherto frequently had to lament their inability to save the child's life."—*British American Journal*, May, 1849.

The above, it will be seen, is announced as a new mode of practice by Dr. McCulloch. By reference to the New York Journal of Medicine for May, 1844, a paper, written by James Couper, M.D., of New Castle, Delaware, will be found, in which a similar practice is recommended, as represented by the following case:

Case.—Mrs. F., having over-exerted herself, was delivered of twins at the end of her eighth month. The labor was slow, but not remarkable in any respect. The first child was a breech presentation, the spine being directed to the left acetabulum of the mother. When the head came to pass the superior strait, the cord was compressed by it, while the expulsive pains became less active than they had been. Soon after this, the pulsation of the cord ceased entirely; the usual convulsive struggle of the child followed; and then all was still. What was to be done in such a case? The spine of an eight months' child would not safely bear the force necessary to an immediate extraction. There was not time for the action of ergot. The face was not yet low enough readily to allow the fingers to be placed at the sides of the nose, that air might have access to the mouth. Nor would the *mere presence* of air have availed, for the child had already become still. In this state of things I executed a plan I had before determined on, and for which I had prepared myself, namely, to *inflate the lungs*, without attempting to extract the child by force. For this purpose I introduced my left forefinger, and with the point of it drew down the chin. Then, using the finger as a director, I passed one end of a long elastic tube, one-fourth of an inch in diameter, well into the mouth, and taking the other end into my own mouth, I blew briskly through the tube two or three times, and immediately had the satisfaction to see respiration established. Muscular efforts followed, and as these stimulated the uterus to stronger contractions, the labor soon came to a happy conclusion. The tube was kept in the mouth of the child until delivery was accomplished.

In commenting upon this case, the then editor, the late S. Forry, M.D., remarks:

"That it is possible for two men to make the same discovery in different ages, or in different countries of the same age, without any knowledge of each other, is sufficiently proved by the fact that, among the ruins of Herculaneum have been discovered surgical instruments, which, thus lost to the world, have been again invented in recent times. The same, it would appear, has happened to our correspondent. In the American Journal of the Medical Sciences, for 1829, Vol. IV., p. 285, is an article by Jacob Bigelow, M.D., Professor of Materia Medica in Harvard University, entitled, '*On the Means of Affording Respiration to Children in Reversed Presentations.*' Referring to the fact that, in those cases of labor in which the body of the child is delivered before the head, many children, which are alive when the body is expelled, are irrecoverably lost in the hands even of the most skilful practitioners, before the head can be extracted; Dr. B. says that the object of his paper is to show that the life of the child, under these circumstances, may often be saved 'by forming a communication between the mouth and the atmosphere, previous to the delivery of the head.'" Dr. Bigelow proceeds:

"The method to be pursued in conveying air to the mouth, depends upon the situation of the head. If the chin has descended low in the pelvis, so that the mouth rests upon the perinæum or lower part of the sacrum, and can be readily reached by the fingers, the hand of the operator alone is sufficient to give the assistance required. But if the mouth is situated so high in the pelvis as to be reached with difficulty, or if, from the large relative size of the head, there is much compression, the assistance of a tube may be of use. The mode of proceeding which I have found successful in various instances, is as follows: as soon as the body and arms are extracted, supposing the face towards the sacrum, an assistant supports the body, carrying it towards the pubis; or the reverse, should the position of the face be to the pubis. The accoucheur should then introduce the hand to which the face looks, till the middle fingers rest upon the mouth of the child. The hand is then to be raised from the throat of the child, making the ends of the fingers a fulcrum, and pushing the perinæum backwards. The air will then pass upwards as far as the chin of the child. The middle fingers are now to be separated about half an inch from each other, and thus a complete passage will be formed between them, by which the air will reach the mouth of the child. If the child be in a healthy state up to this period, it will immediately breathe and cry, and the delivery of the head may be safely postponed until the natural pains recur. If, from any degree of asphyxia, the child does not immediately breathe, it may be often made to do so by dashing cold water upon the body, or by other stimulating processes. It has even appeared to me practicable *to inflate* the lungs, in some cases, *through an elastic catheter.* When the mouth is so high in the pelvis as to be reached with difficulty, or when the compression is so great as to obliterate the cavity between the fingers, a flat tube will be found useful, made of metal, of spiral wire covered with leather, or of elastic gum, and having its largest diameter about half an inch. If the tube be of metal, or of any incompressible material, it should be withdrawn during a pain, to prevent contusion of the soft parts, and immediately replaced, if the pain subsides without expelling the head. Such a tube may be considered as a prolongation of the trachea, and is sufficient to sustain life by respiration for a considerable time. The tube must be guarded and directed by keeping it between the fingers of the inserted hand."

Among the cases given is the following:

"Case V.—After the birth of the body, the face was found so strongly pressed against the sacrum, as to render it difficult to form a passage for the air. By a gentle extractive force, the head was made to descend lower in the pelvis, *and a tube was placed in the mouth.* The child in this situation respired, and, after a few minutes, with the assistance of a pain, the head was easily extracted."

This, it appears, is an old practice, though it seems to have been lost sight of by nearly all our later writers on midwifery. Hence we have deemed the above article worthy of a place on our pages, while its author has all the merit of an original invention.

Dr. Bigelow refers to Pugh's Treatise of Midwifery, published in 1754, which recommends a practice virtually the same.

"'The arms being brought down, the head only remains to be extracted, which must be done with as much expedition as possible, as indeed the arms ought to be; for when the child has passed the navel, the circulation between it and the mother is stopped, from the pressure of the umbilical rope. You must then introduce the fingers of your left hand into the vagina, under the child's breast, and put the first and single fingers into the child's mouth, pretty far, so far, however, that you are able to press down the child's tongue in such a manner that by keeping your hand hollow, and pressing it upon the mother's rectum, the air may have access to the larynx, you will soon perceive the thorax expand, as the air gets into the lungs. Many authors make very little trouble in extracting the head, but without a well formed pelvis, every operator must know there is difficulty and great danger of losing the child by its stay in the passage; but by this method of giving the child air, I have saved great numbers of children's lives, which otherwise must have died.

"'Before I made use of this method, and pressing externally to assist in extracting the head, I found many children were lost in this situation for want of air, which put me upon inventions; as likewise a third, *which was a curved flattish pipe, as likewise a flexible one, that I introduced into the child's mouth as near to the larynx as I could, the other end external,* which I found answer, but now as I find my fingers generally answer, I seldom make use of it.'"—Page 49-50.

As regards the introduction of the fingers into the child's mouth, and thus pressing down the tongue, the direction would seem surperfluous; for, unless the child makes a natural effort to inspire, when the air is brought to its lips, the entire operation will necessarily prove futile. Here Dr. Couper's mode of blowing through the tube would act admirably.—Editor.]

From this it will be seen that the practice recommended by Dr. McCulloch is not original with him, the same idea having occurred to two others previously. In the case related by Dr. Couper, it is very certain that the *mere presence of air* would not have been sufficient.—Eds. Medical Examiner.]

